# Isolation and growth of neural stem cells derived from adult human hippocampus

**Published:** 2012-11

**Authors:** Mousa Taghipour, Ali Razmkon

**Affiliations:** ^a^Shiraz Neurosciences Research Center, Shiraz University of Medical Sciences, Shiraz, Iran.

## Abstract

**Background:**

The hippocampus is known to be required in the processing of spatial and contextual memories. A specific role in this context appears to be played by the new neurons continuously generated during adulthood from progenitor cells in the subgranular zone of the dentate gyrus. Ablation of neurogenesis by toxins, x-ray irradiation or virus-activated pro-drugs causes severe cognitive deficits in mice models.

**Methods:**

Histologic specimens from adult human hippocampus, dissected during the amygdalohippocampectomy process for mesial temporal sclerosis, were obtained and cultured in suitable media. Neural stem cells were characterized using an appropriate in-vitro molecular biomarker profiling.

**Results:**

A total of six adult human hippocampal tissues were evaluated of which, two specimens retrieved well-characterized neural stem cells.

**Conclusions:**

The adult mammalian hippocampus contains neural precursor cell populations, which give rise to new neurons by passing a sequence of different amplifying lineage-determined progenitor cells. While some of the newborn neurons are integrated into the functional circuitry, most of them have only a transient existence. These cells provide the substrate for neuronal plasticity at the cellular level eventually playing a role in learning and memory.

**Keywords:**

Neural stem cell, Cell cultutre, Hippocampus, Neural stems cells


					Volume 4, Suppl. 1 Nov 2012
Publisher: Kermanshah University of Medical Sciences
URL: http://www.jivresearch.org
Copyright:
Congress of Iranian Neurosurgeons, 14-16 November 2012, Kermanshah, Iran
Paper No. 18
Isolation and growth of neural stem cells derived from adult
human hippocampus
Mousa Taghipour a
, Ali Razmkon a,*
a
Shiraz Neurosciences Research Center, Shiraz University of Medical Sciences, Shiraz, Iran.
Abstract:
Background: The hippocampus is known to be required in the processing of spatial and contextual memories. A
specific role in this context appears to be played by the new neurons continuously generated during adulthood from
progenitor cells in the subgranular zone of the dentate gyrus. Ablation of neurogenesis by toxins, x-ray irradiation or
virus-activated pro-drugs causes severe cognitive deficits in mice models.
Methods: Histologic specimens from adult human hippocampus, dissected during the amygdalohippocampectomy
process for mesial temporal sclerosis, were obtained and cultured in suitable media. Neural stem cells were
characterized using an appropriate in-vitro molecular biomarker profiling.
Results: A total of six adult human hippocampal tissues were evaluated of which, two specimens retrieved well-
characterized neural stem cells.
Conclusions: The adult mammalian hippocampus contains neural precursor cell populations, which give rise to new
neurons by passing a sequence of different amplifying lineage-determined progenitor cells. While some of the
newborn neurons are integrated into the functional circuitry, most of them have only a transient existence. These cells
provide the substrate for neuronal plasticity at the cellular level eventually playing a role in learning and memory.
Keywords:
Neural stem cell, Cell cultutre, Hippocampus, Neural stems cells
* Corresponding Author at:
Ali Razmkon: Shiraz Neuroscience Research Center, Shiraz University of Medical Sciences, Shiraz, Iran, Tel:09173148711,
Email: ali.razmkon@gmail.com, (Razmkon A.).

				

